# Healthcare-Associated Infections in a Tertiary Care Hospital: Significance of Patient Referral Practices

**DOI:** 10.24248/eahrj.v8i1.759

**Published:** 2024-03-28

**Authors:** Agapiti H. Chuwa

**Affiliations:** a University of Dar Es Salaam, Mbeya College of Health and Allied Sciences, Mbeya, Tanzania

## Abstract

**Introduction:**

Nosocomial infections, also known as healthcare-associated infections (HCAIs), are infections that a patient acquires while receiving healthcare services within 48 hours of admission to hospital. In high income countries, an average of 7% of hospitalised patients acquire a nosocomial infection. In low and middle income countries, however, prevalence rates can be as high as 30%. This is due to limited resources and supplies for infection control, inadequate training and lack of compliance with infection prevention and control regulations.

**Methods:**

A cross-sectional, hospital based study was conducted among patients admitted to a tertiary care facility in Tanzania. A semi-structured questionnaire was used to collect information from 134 patients in different wards. Correlation and multivariate regression analyses were performed to determine the association between the independent variables, i.e. chronic illness, invasive procedures, use of antibiotics and referral status, and the dependent variable, i.e. new symptom, and their level of significance. The significance level was set at *P≤ .05*.

**Results:**

A total of 134 patients participated in the study. Of the total number, 61% (n=82) of the patients were male and 43% (n=57) were referrals from other institutions. Fifteen percent (n=21) of the surveyed patients reported a new symptom. There was a positive correlation between referral status and invasive procedure with the occurrence of a new symptom. Multivariate analysis identified ‘referral status' as an independent significant factor positively associated with healthcare-associated infetions (*P=.041*).

**Conclusions:**

The results indicate a prevalence of 15.7% of healthcare-associated infections, which is unacceptably high for a tertiary care facility. Referral status was independently and significantly associated with HCAI. Improving patient referral patterns and hospital infection control can significantly reduce the risk of healthcare-associated infections.

## BACKGROUND

Healthcare-associated infections (HCAIs), also known as nosocomial infections or hospital-acquired infections (HAIs), are infections caused by bacteria, viruses or fungi that are acquired by a patient within 48 hours of admission to a healthcare facility, up to three days after discharge, or up to 30 days after surgery. Such infections are acquired by a patient while receiving healthcare services, i.e. diagnostic, therapeutic and preventive services. HCAIs are associated with the emergence of multidrug-resistant microorganisms. This is a factor that leads to a significant burden of morbidity, mortality and associated costs for patients and their families.^1, 2^ They are classified as catheter-associated urinary tract infections, central line-associated bloodstream infections, ventilator-associated pneumonia and surgical site infections (SSIs).^3^ Proposals for the definition and classification of healthcare-associated infections were first put forward in 2002 by Siegman-Igra et al and Friedman et al.^4, 5^ However, the definition of healthcare-associated infections is highly variable. Despite the adoption of several alternative definitions, the original statement by Friedman et al is still widely used in clinical research.^5, 6–9^

Healthcare associated infections are very unevenly distributed around the world. In high-income countries, approximately 15% of hospitalised patients and up to 37% of patients admitted to intensive care units develop HCAIs.^1, 6,7^ The pooled prevalence of HCAIs in Africa varies from 14.2% to 23.2%.^8, 9^ The average prevalence of HCAIs is 7% in high-income countries and 10% in llow-and-middle income countries (LMICs). In the latter, however, it can be as high as 50% in patients admitted from the community.^5, 10–13^ Inadequate resources, lack of infection prevention and control (IPC) training, and non-compliance with standard operating procedures are among the major causes of healthcare-associated infections in LMICs. In Ugandan hospitals, for example, an assessment of the role of medical devices in healthcare associated infections found that 9 out of 10 devices tested positive for contamination in at least 1 location and 2 out of 3 devices tested positive in at least two or more locations.^14^ The 3 most commonly isolated microorganisms were Bacillus species (53%), coagulase-negative staphylococci (15%) and *Escherichia coli* (13%). More worryingly, almost 30% of samples were resistant to 3 or more classes of antibiotics tested, including penicillin, tetracycline, glycylcycline and trimethoprim-sulfamethoxazole. Another study found that 93.4% of health facilities surveyed (30/32) did not have adequate infection control equipment or supplies.^15^ There were also reports of inadequate funding, training and low compliance with IPC.^16^ Similar factors have been blamed for high rates of healthcare-associated infections in other African countries.^17–20^

Risk factors for HCAIs include a history of surgery or other invasive procedures, active or metastatic cancer, immunosuppression due to prolonged use of steroids, human immunodeficiency virus (HIV)/acquired immune deficiency syndrome (AIDS), radiotherapy and other causes, and transfer from another healthcare facility. The relationship between healthcare-associated infections and patient transfer patterns is only partially understood. In their study, Xia H. et al. argue that direct patient transfers between hospitals have only a limited impact on healthcare-associated infections and that readmission to the same hospital poses the greatest risk.^21^ Other reports show that reducing the number of transfers by diverting an average of 1.5 patients per hospital per day could reduce the spread of high-risk bacterial clones in hospital networks by 36%.^22, 23^ In contrast, another study found that patient diversion strategies alone could not reduce the prevalence of multidrug-resistant *Enterobacteriaceae* (MDR-E) at the level of a regional healthcare network.^24^ Targeted hospital-based interventions focused on hospitals identified by network transmission patterns could reduce the spread of MDR-E by half.^24^ There are currently no reports on the role of patient referral patterns in healthcare-associated infections in Africa. The current study sought to identify factors influencing healthcare-associated infections in a tertiary referral hospital in Tanzania, taking into account patient referral patterns. The results of this study contribute to better patient referral practices and open new avenues for investigating the components of referral patterns that are specifically associated with HCAIs.

## METHODS

### Study Area

The current study was conducted in a 553-bed tertiary health facility serving a population of over ten million people in Tanzania. The hospital is the only highly specialised health facility in the region and a referral centre for patients from eight administrative regions.

### Study Design

A hospital-based cross-sectional study was conducted in a tertiary teaching hospital in Tanzania from July to August 2023. Patients from different wards - surgical, maternity, medical, E.N.T, intensive care and paediatric were randomly selected. The sample size was determined using Fisher's formula N= (Z2 x p(1-p))/e2 where N=number of minimum sample size, p=prevalence of nosocomial infections among inpatients. The recently reported point prevalence of HCAI in LMICs ranges from 7.67% to 12.76%.^10, 11^ The average prevalence of 10% reported by the World Health Organization was used in this study.^11^ Z= normal standard deviation with 95% confidence interval, i.e. 1.96, and e= standard maximum error, i.e. 0.05. A total of 134 eligible patients (patients admitted for more than 48 hours at the time of the study) participated in the study. Only time, person and place-oriented patients who were not critically ill were included in the study.

### Data Collection

Data were collected using a semi-structured questionnaire, interviews and observational methods. Patients were given questionnaires translated into Swahili and asked to answer questions about length of hospital stay, invasive procedures, referral status, occurrence of a new symptom (fever, pus in the wound, cough, urinary symptoms) and use of antibiotics. The investigators supervised and observed the completion of the questionnaires and compared the patients' fever curves and other records with their responses.

### Statistical Analysis

The data obtained were entered and analysed using MS Excel and SPSS (Statistical Package for the Social Sciences, IBM Corp., NY, USA) version 23. Categorical variables were expressed as numbers and percentages. Correlation and multivariate regression analyses were performed to determine the association of the independent variables, i.e. chronic disease, invasive procedures, antibiotic use and referral status, with the dependent variable, i.e. new symptom, and their levels of significance. The significance level was set at *P≤ .05*.

### Ethical Approval and Informed Consent

Approval to conduct the study was granted by the Ethical Clearance Sub-Committee (ref. AB 458/482/02/476) of the University of Dar es Salaam, Mbeya University of Health and Allied Sciences. Informed consent was also obtained from all study participants. Interviews were conducted separately and responses were recorded anonymously.

## RESULTS

### Socio-demographic Characteristics of Participants

One hundred and thirty-four patients participated in the study and all (100%) answered all questions. Of the participants, 61%(82/134) were male. The majority of patients, 57%(76/134), were aged between 18 and 45 years. Of the patients surveyed, 43%(n=57) were referred from other institutions, while 57%(n=77) were directly admitted, (Table 1).

**TABLE 1:** Socio-demographic Characteristics of the Study Participants

Variable	Frequency (N)	Percentage (%)
Sex		
Male	82	61
Female	52	39
Age (Years)		
<18	47	35
18–45	76	57
45–60	4	3
>60	7	5
Referral status		
Referred from other facilities	57	43
Direct admission	77	57

### Common Symptoms of Nosocomial Infections

**FIGURE 1:**
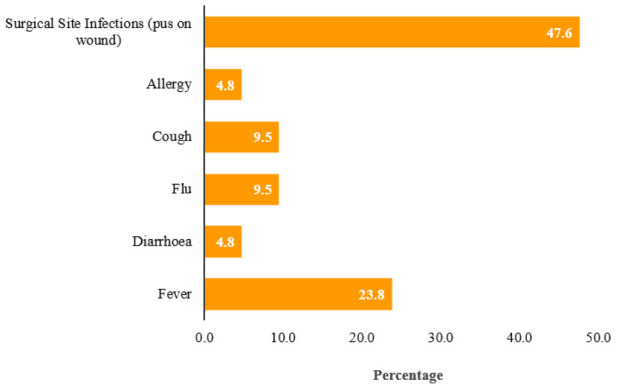
Prevalence of common symptoms of Healthcare-Associated Infections

Of all respondents, 15.7%(n=21) reported a new symptom after hospital admission. The most commonly reported new symptoms were surgical site infections; presented as pus on the wound 47.6%(n=10), fever 23.8%(n=5); confirmed from patient temperature records, cough 9.5%(n=2), rhinitis 9.5(n=2), diarrhoea 4.8%(n=1) and pruritus 4.8%(n=1), (Figure 1).

### Determinants of Nosocomial Infections

To gain further insight into the determinants of the development of new symptoms after admission, data on the categorical variables; chronic illness, use of antibiotics, invasive procedure and referral status were coded, analysed and tested against a dependent variable; new symptom. A Pearson correlation analysis showed that the variables ‘referral status' and ‘invasive procedure' tended to correlate positively with the occurrence of a new symptom (Table 2). Multivariate regression analysis showed that ‘referral status' had a significant (*P=.047*) positive association with the occurrence of a new symptom (Table 3). Chronic illness and antibiotic use, although not statistically significant, showed a tendency to be negatively associated with the development of new symptoms. Apart from referral status, all other variables had significance levels *P>.15* and were therefore dropped. Regression of the variable ‘referral status' against the development of a new symptom resulted in a significance level of *P=.041*.

**TABLE 2:** Correlation Between Independent Variables and the Occurrence of a New Symptom

	Use of Antibiotics	Referral	Invasive procedure	Chronic illness	New symptom
Use of antibiotics	1				
Referral	−0.0622	1			
Invasive procedure	0.0472	−0.0406	1		
Chronic illness	0.0706	−0.0375	−0.0432	1	
New symptom	−0.0575	0.1770	0.0492	−0.1207	1

**TABLE 3:** Significance Levels of the Categorical Variables with the Occurrence of a New Symptom

	*Coefficients*	*Standard Error*	*t Stat*	*P-value*	*Lower 95%*	*Upper 95%*
Intercept	1.0829	0.2116	5.1188	0.0000	0.6643	1.5015
Use of Antibiotics	−0.0723	0.1502	−0.4817	0.6309	−0.3695	0.2248
Referral	0.1032	0.0516	2.0018	0.0474	0.0012	0.2053
Invasive procedure	0.0326	0.0525	0.6209	0.5358	−0.0713	0.1365
Chronic illness	−0.0995	0.0787	−1.2637	0.2086	−0.2552	0.0563

## DISCUSSION

Healthcare-associated infections represent a significant burden for patients, families and healthcare systems. The results of the current study show that the prevalence of healthcare-associated infections in the tertiary healthcare facility surveyed was 15.7%. Similar results were observed in a study conducted in another tertiary hospital in northern Tanzania (prevalence rate of 14.8%), also consistent with results from other countries in East Africa.^17–20, 25^ In contrast to previous studies, the current study shows for the first time in an African country that patient referral patterns have a major impact on the transmission of HCAIs. Within the health care system, patients are referred to hospitals at different rates depending on the function and level of the hospital. This is likely to affect the prevalence of healthcare associated infections in different institutions.

It was also found that 10 out of 21 patients with new symptoms reported the presence of pus in the wound, which represents 7.5% of the patients surveyed. This is in line with another study conducted in Tanzania which found that 10.6% of patients surveyed (n=128) had a surgical site infection.^26^ In general, the incidence of surgical site infections in sub-Saharan Africa ranges from 6.8% to 26%.^27^ Surgical site infections are associated with prolonged hospital stay and increased risk of mortality. To reduce the burden of SSIs, the World Health Organization technical team developed the Global Guidelines for the Prevention of Surgical Site Infections in 2018, available at https://iris.who.int/bitstream/handle/10665/277399/9789241550475-eng.pdf?sequence=1. In summary, the results of the current study highlight the high prevalence of healthcare-associated infections in Tanzania and the importance of improving interventions to prevent surgical site infections. In addition, the results demonstrate for the first time in Africa the association between patient referral patterns and HCAIs.

## CONCLUSIONS AND RECOMMENDATIONS

The results indicate a high prevalence of healthcare-associated infections in a tertiary referral hospital in Tanzania. I, therefore recommend an integrated and efficient approach that includes monitoring referral hospital linkages with other health facilities to improve patient referral patterns, and hospital infection control to reduce healthcare-associated infections.

### Study Limitations

Although the current study found that healthcare-associated infections were more common in patients referred from other healthcare facilities, the study did not examine compliance with IPC rules by hospital staff. Bacteriological investigations and further research into factors related to patient referral behaviour were also not carried out, which would warrant a follow-up study.
